# BFF: Bayesian, Fiducial, and Frequentist Analysis of Cognitive Engagement among Cognitively Impaired Older Adults

**DOI:** 10.3390/e23040428

**Published:** 2021-04-06

**Authors:** Shevaun D. Neupert, Claire M. Growney, Xianghe Zhu, Julia K. Sorensen, Emily L. Smith, Jan Hannig

**Affiliations:** 1 Department of Psychology, North Carolina State University, Raleigh, NC 27695, USA; xzhu23@ncsu.edu (X.Z.); jksorens@ncsu.edu (J.K.S.); ejlefebv@ncsu.edu (E.L.S.); 2 Department of Psychological and Brain Sciences, Washington University in St. Louis, St. Louis, MO 63130, USA; cgrowney@wustl.edu; 3 Department of Statistics and Operations Research, University of North Carolina at Chapel Hill, Chapel Hill, NC 27599, USA; jan.hannig@unc.edu

**Keywords:** cognitive engagement, cognitive impairment, frequentist, Bayesian, fiducial paradigm

## Abstract

Engagement in cognitively demanding activities is beneficial to preserving cognitive health. Our goal was to demonstrate the utility of frequentist, Bayesian, and fiducial statistical methods for evaluating the robustness of effects in identifying factors that contribute to cognitive engagement for older adults experiencing cognitive decline. We collected a total of 504 observations across two longitudinal waves of data from 28 cognitively impaired older adults. Participants’ systolic blood pressure responsivity, an index of cognitive engagement, was continuously sampled during cognitive testing. Participants reported on physical and mental health challenges and provided hair samples to assess chronic stress at each wave. Using the three statistical paradigms, we compared results from six model testing levels and longitudinal changes in health and stress predicting changes in cognitive engagement. Findings were mostly consistent across the three paradigms, providing additional confidence in determining effects. We extend selective engagement theory to cognitive impairment, noting that health challenges and stress appear to be important moderators. Further, we emphasize the utility of the Bayesian and fiducial paradigms for use with relatively small sample sizes because they are not based on asymptotic distributions. In particular, the fiducial paradigm is a useful tool because it provides more information than *p* values without the need to specify prior distributions, which may unduly influence the results based on a small sample. We provide the R code used to develop and implement all models.

## 1. Introduction

Using multiple statistical paradigms can be helpful for examining the robustness of effects. We seek to rigorously apply frequentist, Bayesian, and fiducial statistical paradigms to identify factors that may contribute to cognitive engagement for individuals who are already experiencing cognitive decline. Engaging in cognitively demanding activities is beneficial in preserving cognitive health. Selective engagement theory [1] argues that normative increases in the cognitive costs associated with demanding activities reduce older adults’ motivation to engage in those activities and lead to greater selectivity in participation in behaviors that put demands on cognitive resources. However, selective engagement theory has not been examined with cognitively impaired older adults and it has been exclusively tested within the frequentist statistical paradigm.

One reasonable expectation based on selective engagement theory [1] is that disproportionate increases in cognitive costs contribute to decreased motivation, which in turn leads to selectivity in cognitive engagement. That is, older adults with cognitive impairments may disengage from tasks they view as costly to a greater extent than older adults with normative patterns of cognitive change. For example, sample, approximate, and spectral entropy as well as Lempel-Ziv and Lopez Ruiz-Mancici-Calbet complexity tend to be lower in Alzheimer’s patients, corresponding to more predictable and consistent electroencephalogram (EEG) and magnetoencephalography (MEG) signals [2]. This lower entropy and complexity may be associated with disengagement. It is possible that older adults with cognitive impairment lack the cognitive resources involved in weighing the potential costs versus benefits of engaging in a particular task. Therefore, they may be relatively less discerning when selecting activities, and incur large cognitive costs as a result. 

Systolic blood pressure responsivity (SBP-R) is a useful index of engagement or required costs (e.g., [3,4]), and has been positively associated with objective levels of task difficulty in cognitively healthy older adults (e.g., [5,6]). SBP-R may reflect cardiovascular health, cardiovascular reactivity to stress, or cardiovascular responsivity to cognitive engagement [7]. Further, effortful engagement as indexed by SBP-R is associated with motivation to engage (e.g., [3,8]). There are also age differences in SBP-R during various laboratory tasks, with older adults demonstrating higher reactivity (for review, see [9]), which according to the Strength and Vulnerability Integration model [10] may present an obstacle to older adults’ information processing. In cognitively healthy older adults, responsivity requires active coping with the demands of laboratory-based cognitive tasks [11]. It is less clear, however, the extent to which cognitively impaired individuals engage in active coping when exposed to cognitive stressors. Although aging is associated with predictable increases in SBP-R (e.g., [3]), higher cognitive costs, the experience of which are common in impaired older adults, are associated with lower motivation and engagement (e.g., [3,12]). In addition to cognitive resources, an examination of physical and mental health resources and stress levels as potential moderators is a crucial component to our understanding of SBP-R in cognitively impaired older adults. 

### 1.1. Physical and Mental Health

Physical and mental health have important influences on cognitive processes. Better physical health is associated with better memory performance in midlife [13], and worse physical health and more depressive symptoms predict lower as well as steeper declines in cognitive functioning longitudinally [14]. Therefore, physical and mental health can function as important resources for cognition within cognitively normal adults [15], and we are interested in the role of cognitive and mental health challenges experienced by cognitively impaired older adults. In addition, we examine the potential role of longitudinal changes in cognitive and mental health challenges.

### 1.2. Stress

Persistent or chronic stress may exacerbate the negative effects of aging on SBP-R [7] and is detrimental for cognitive functioning [16]. Prolonged stress across age is linked with impaired cognitive functioning and performance [17], and one of the best ways to assess prolonged stress is through assays of hair [18]. Hair cortisol concentration (HCC) provides a retrospective view of the hypothalamic-pituitary-adrenal axis [19]. 

We examine the interplay of SBP-R and stress within a cognitively impaired older adult sample using a longitudinal design. Specifically, we examine stress (hair cortisol) as well as physical and mental health challenges as potential moderators between cognitive ability and engagement and also investigate longitudinal change in these factors as moderators. Evaluating the presence or absence of effects is a core scientific challenge that cuts across disciplines. Lakens et al. [20] described Bayes factors as useful tools to improve inferences about null effects within gerontology beyond frequentist statistics, and Neupert and Hannig [21] demonstrated the utility of generalized fiducial inference for detecting age-related similarities and differences. Our methodological innovation is the application of multilevel models from three different statistical paradigms to enable clear determinations about effects.

### 1.3. Frequentist Paradigm

The frequentist paradigm is the framework in which well-known methodologies of statistical hypothesis test, *p*-values, and confidence intervals are based. It is a type of statistical inference that draws conclusions from sample data by emphasizing the frequency or proportion based on the sampling distribution of the data. However, it has a number of weaknesses. Inference is typically based on an assumption of asymptotic distribution which might not work well for small sample sizes [21]. The use of *p*-values has also come under fire because of the arbitrary cut-offs [22]. Importantly, when *p*-values are larger than the arbitrary cut-off, it is not appropriate to definitively conclude a null effect [21].

Despite these weaknesses, theoretical principles within aging have been built on studies using the frequentist paradigm. Specifically, selective engagement has been investigated by Smith and Hess [23] and Hess and colleagues [3] who found systematic increases in SBP which aligned with objective task demands, demonstrating that the costs of cognitive engagement increase as task demands increase. Further, the experience of relatively high costs during cognitive engagement has been associated with lower levels of motivation to engage, which in turn predicts actual engagement in everyday life [3]. Both trait-level and situation-specific motivation have been identified as key predictors of task engagement as indexed by SBP-R as well [4], with higher motivation predicting higher SBP-R. Costs associated with task engagement have also been associated with willingness to continue engaging in similarly difficult tasks, with high costs disproportionately limiting older adults’ motivation to engage [24]. Importantly, Hess, Emery, and Neupert [15] found that individual differences in health, as well as changes in health over time, were important resources that predicted changes in motivation, which in turn was associated with involvement in cognitive activities and level of cognitive ability. They also found that the impact of declining health on motivation was particularly strong in older adulthood, but their sample was cognitively intact. While model comparison using methods like AIC, BIC or Mallows *C_p_* are available, they are not commonly used within the cognitive aging literature. We extend previous studies that examined normative aging processes within the frequentist paradigm to cognitively impaired older adults across three statistical paradigms to rigorously test principles from selective engagement theory [1].

### 1.4. Bayesian Paradigm

The Bayesian paradigm uses probability more widely than the frequentist paradigm to model both sampling and other kinds of uncertainty. The critical conceptual difference between the frequentist and Bayesian paradigms is the interpretation of the meaning of probability. Bayesian inference requires selecting a model and a prior distribution (i.e., a probability distribution summarizing prior knowledge about unknown parameters) that is personalized or subjective [25]. The result of a Bayesian approach is a probability distribution conditional on the sample data called the posterior. Inference is then based on the posterior and does not target repeated sampling properties based solely on sampling distributions. The Bayesian method does not rely on an asymptotic distribution and all models are treated equally, which is in contrast to the frequentist paradigm where the null model has special significance. A critical weakness of the Bayesian method is the requirement to select a prior even when no prior information is available, which is problematic because: (1) conclusions are dependent on which prior is selected [26]; and (2) the uniform LaPlace prior that is often recommended when there is dearth of prior information is not always appropriate [27].

Instead of *p*-values, models are often assessed using Bayes factors [28]. Bayes factors compares the strength of evidence between pairs of models and allows us to find the model that is most in agreement with our data and priors. For example, Bayes factors can be used to select between pairs of models that test whether there is evidence that cortisol has an effect on cardiovascular responsivity. Bayes factors allow us to quantitatively compare the strength of evidence for the various models. Important predictors are then found by comparing the relative strength of evidence between the best model and other models using the Bayes factors. This is different from the classical frequentist paradigm as used in cognitive aging research, in which we find important predictors by fitting the full model and looking for evidence of coefficients not being equal to zero using *p*-values. Bayes factor measures the strength of evidence for one model over another, regardless of whether these models are correct. Although the Bayes factors compare pairs of models which can make for clear decisions regarding which model is a better fit, they do not provide a direct way of testing if either model is actually a good fit [21]. That is, the pairwise comparisons help researchers make decisions about which model is better, but it is possible that neither model is good.

Although there are widely accepted likelihood models, such as the multilevel model used in the present study, prior distributions are much more difficult to select [21]. This difficulty is compounded by the fact that prior selection will affect the outcome [26]. However, there are tools that allow researchers to check whether their model and prior is in conflict with the data [29,30,31,32].

### 1.5. Fiducial Paradigm

The origin of fiducial inference can be traced back to Fisher [33] who introduced the concept of a fiducial distribution for a parameter, and proposed the use of a fiducial distribution in place of the Bayesian posterior distribution. In simple situations, especially in one parameter families of distributions, fiducial intervals coincide with classical confidence intervals. For multi-parameter families of distributions, the fiducial approach led to confidence sets whose frequentist coverage probabilities were close to the claimed confidence levels but they were not exact in the repeated sampling frequentist sense. This led to major discussions among the prominent statisticians of the mid 20th century [34]. Many of these discussions focused on the non-exactness of the confidence sets, non-uniqueness of fiducial distributions, and other issues with coherence.

Generalized Fiducial Inference is a modern reincarnation that capitalizes on the strengths and advances made in both frequentist and Bayesian methods during the last century, such as MCMC. The main idea of fiducial inference is to define a distribution for parameters of interest that captures all of the information that the data contain about these parameters, but without assuming a prior distribution [35]. Whereas a Bayesian approach starts with a fully specified, single joint probability distribution and then predicts a value using conceptually simple probabilistic computations (Bayes theorem), the fiducial approach is similar to the frequentist approach in the modeling step because it considers a number of potential distributions for the observed data as the model [35]. Like the frequentist paradigm, the fiducial paradigm also assumes that the hypothesis is either true or not, i.e., it does not assume that the parameter is random. However, the fiducial paradigm provides a probability distribution based on the observed data. This is achieved by selecting a model equation (sometimes called data generating algorithm) that links parameters, auxiliary random variables (usually having standard normal distribution) and the data (see the analysis section for some examples). The fiducial distribution is then obtained by transferring randomness from the auxiliary space to the parameter space using an inverse of the model equation without the use of Bayes theorem [36].

Though Generalized Fiducial Inference is relatively new and is less familiar than the frequentist paradigm, there are already several examples when its confidence intervals are better than any other available in the literature (e.g., [21,37,38]). The fiducial paradigm provides a compromise between a posterior-like distribution on a parameter which allows for uncertainty quantification, similar to Bayesian, but it produces inference with good frequentist properties (i.e., confidence intervals) without the need to derive an asymptotic theorem. One of its limitations is the fact that in many practical situations the fiducial distribution depends on the data generating algorithm which introduces potential incoherence. This dependence on the data generating algorithm means that like the frequentist paradigm, the fiducial paradigm does not follow the likelihood principle. Furthermore, because it does not allow for the use of conjugate priors, computations of the fiducial distribution are often more complicated than its Bayesian counterpart.

We recently developed and applied Generalized Fiducial Inference to an investigation of age differences in emotional reactivity to daily stressors with a daily diary design [21]. We compared results from frequentist, Bayesian, and fiducial paradigms and found convergence across four of the six models, concluding evidence of null age effects in reactivity. In one model, however, the frequentist model suggested an age difference where the fiducial model did not, and in another model the fiducial result suggested an age difference where the frequentist result did not. Thus, we had more confidence in the results where all three paradigms agreed and concluded strong evidence of null age effects in those models.

### 1.6. Present Study

Our overarching aim is to apply multilevel models using frequentist, Bayesian, and fiducial paradigms to identify predictors of SBP-R during task engagement in a cognitively impaired sample of older adults. In doing so, we will demonstrate the utility of these statistical methods for evaluating the robustness of developmental phenomena. In the present longitudinal study, we examine SBP-R of cognitively impaired older adults during a cognitive task. In addition to considering cognitive ability, we examine physical (Research Question 1) and mental health challenges (Research Question 2) as well as long-term stress (Research Question 3) as potential moderators. In addition, we examine longitudinal changes in physical (Research Question 4) and mental health challenges (Research Question 5) as well as long-term stress (Research Question 6) as moderators of cognitive ability differences in SBP-R over time. For each of these research questions, we will compare the results across the three statistical paradigms and place more confidence in results where all of the paradigms agree. We will provide recommendations and considerations for practitioners when results are divergent across paradigms.

## 2. Materials and Methods

### 2.1. Participants

Participants in the longitudinal study were adults age 61–85 who were either diagnosed with a cognitive impairment such as dementia or failed the Short Blessed Orientation-Memory-Concentration Test [39] with a score of 7 or higher.

### 2.2. Measures

Self-report measures included physical and mental health challenges (see Appendix A). Cortisol was collected via hair samples (see Appendix A) and consistent with previous work [20], we conducted a log transformation of the HCC values for analysis. Cognitive ability was determined through a series of cognitive assessments that were combined to create a factor score with higher values indicating higher cognitive ability, or less cognitive impairment (see Appendix A).

#### Cognitive Engagement and Cardiovascular Monitoring

Participants completed a computerized memory-scan task in which they were presented with a string of consonants on the screen followed by a single consonant. They were instructed to indicate whether or not the single consonant was presented in the string of consonants viewed immediately before by indicating “Yes” or “No” using a serial response box. The task was programmed in E-Prime and had four levels of increasing difficulty, with 30 items per level consisting of one, three, five, and seven letters, respectively. As participants are required to sustain engagement throughout each level of the task, task difficulty increases as a result of expended resources. Thus, we divided each level into three tertiles representing the beginning, middle, and end of each level, resulting in 12 tertiles of increasing difficulty at each wave. The use of tertiles also allowed us to use the same number of observations within each participant, because the length of time to complete the task could vary between participants.

While completing these tasks, cardiovascular responsivity was assessed using a continuous noninvasive arterial blood pressure device, CNAP Monitor 500 HD (CNSystems Medizintechnik AG, Graz, Austria). The device incorporates two finger cuffs placed around the index and middle fingers which continuously inflate and capture beat-by-beat finger arterial pressure. These measurements are calibrated with brachial arterial pressure through a standard BP measurement cuff around the upper arm. A BIOPAC MP150 system (BIOPAC Systems, INC., Goleta, CA, USA) was used to transfer cardiovascular data and temporal information from the E-Prime task to a separate computer which recorded all data using AcqKnowledge software.

### 2.3. Procedure

As a part of a larger study, participants followed the same general procedure at both waves of assessment, which occurred approximately six months apart.

After confirming eligibility and giving informed consent, participants completed a series of questionnaires about their abilities, attitudes, and well-being, including the Geriatric Depression Scale. They received these questionnaires through email to be completed at home, with the option to complete them with their proxy. Emails were sent directly to the proxy for those unable to use email independently.

Participants came into the lab for the next portion of the study. They first completed the Community Screening Instrument -Dementia and Harmonized Cognitive Assessment Protocol tasks administered by a researcher. The researcher then collected hair samples from the participant. We followed collection procedures for short (<3 cm) and long (>3 cm) hair sampling detailed by Stalder and Kirschbaum [18] and by Kirschbaum’s lab (http://www.dresden-labservice.de/, accessed on 19 June 2019).

After being connected to the CNAP monitor, participants sat and relaxed silently for 10 min while cardiovascular data were collected. The last 5 min of this time period was used to represent baseline cardiovascular responsivity. To increase motivation to engage in the cognitive tasks, participants were presented with a message that the computer would keep track of their performance and they would discuss their performance with the researcher. All information and instructions which appeared on the screen were also read aloud by the researcher. Before beginning the task, participants completed some practice trials to become familiarized with the setup. Participants completed four levels of the 30-item memory scan task while connected to the CNAP monitor, as well as some additional surveys and tasks which will not be discussed in the present paper. Researchers reminded participants about task instructions and provided additional guidance as needed.

At the end of the session, participants completed a series of questionnaires about their health including the SF-36 and chronic conditions checklist. They had the option to complete these questionnaires with their proxy, if applicable. Finally, participants received compensation for their time and efforts.

## 3. Analysis

### 3.1. Data Preparation

SBP-R and baseline SBP were computed using SAS [40]. Participants varied in the amount of time spent completing each level of the task and had one measure of SBP per second. As previously described, the tertiles varied in length both between and within individuals by dividing the number of seconds spent completing each level by three. We then averaged the second-by-second SBP values within each tertile. These values were converted to SBP-R by subtracting baseline SBP values. These preparation procedures resulted in up to 24 rows of data for each participant (2 waves * 12 tertiles per wave), yielding a total of 504 observations for analysis. Results from an initial unconditional model testing whether there was systematic variance in SBP-R between the two waves suggested that there was not (estimate of wave-level random effect variance: τ_00_ = 27.88, *SE* = 28.92, *p* = 0.168), so the analyses treat the tertiles (Level 1) as nested within persons (Level 2).

### 3.2. Modeling

We conducted six multilevel models (MLM) [41]) within the frequentist paradigm. A multilevel model is a linear random effects model that is organized so that the nested structure within the data is easily understood. Level 1 represents all of the repeated measures data and uses covariates independent of the individual participants such as time (i.e., tertile). The Level 1 coefficients are themselves modeled on Level 2 as linear functions of individual covariates such as physical and mental health measures that are particular to each person. Because errors are introduced on both levels, this creates correlated overall errors leading to a particular linear mixed model. An example of such a model formulation that is common in the cognitive aging literature and used to evaluate Research Question 1 in this paper follows:
Level 1 (within-person, *t*):  SBP-R_ti_ = ß_0ti_ + ß_1ti_(Tertile) + r_ti_Level 2 (between-person, *i*):  ß_0i_ = γ_10_ + γ_01_(SBP baseline) + γ_02_(Cognition) + γ_03_(Physical health challenges) + u_0i_  ß_1i_ = γ_10_ + γ_11_(Cognition) + γ_12_(Physical Health Challenges)

The within-person (Level 1) intercepts (ß_0_) and slopes (ß_1_) become the outcomes at the person level (Level 2). The within-person time effect was indexed by tertiles within tests (γ_10_). Main effects of SBP at baseline (γ_01_), cognition (γ_02_), and physical health challenges (γ_03_) were included as predictors of the intercept. The Cognition × Tertile (γ_11_) cross-level interaction tests for cognition differences in changes in SBP-R. The Physical Health Challenges × Tertile (γ_12_) cross-level interaction tests for physical health challenge differences in SBP-R. The random intercept (u_0i_) was allowed to vary across persons and was assumed to follow a multivariate normal distribution with a mean of zero and covariance matrix ∑. The residual error r_ti_ is independent of the random intercept and follows a normal distribution with a mean of zero and variance *σ*^2^. Notice that technically speaking only the *γ*s, *σ*^2^, and ∑ are the parameters of this model.

Multilevel analyses within the Bayesian paradigm included the same fixed and random effects as in the frequentist analyses. However, there are some notable differences. Bayesian analysis tests for the differences between pairs of models, so the pairs need to be specified. In addition, the key in applying the Bayesian paradigm is the appropriate selection of priors for parameters. A set of principles for prior selection in the context of model selection has been proposed by Bayarri, Berger, Forte, and García-Donato [42]. In this paper, we use a modification of the Zellner and Siow [43] as described in Rouder, Morey, Speckman, and Province [44] which is implemented in R package BayesFactor.

Model comparisons were done using Bayes factors [20]. Bayes factors are computed as the ratio of the marginal distribution of the data computed under each of the models. Specifically, we calculated Bayes factors (K) to quantify support for comparisons between all possible combinations of fixed effects against an intercept only (null) model. This essentially functions like an all-subsets regression, but the intercept was always included to reduce the number of potential models. Jarosz and Wiley [45] review guidelines on the use of Bayes factor. In particular, they report that ratios of K values larger than 10 or 20 are generally considered strong evidence in favor of the better fitting (larger K) model. On the other hand, Baskurt and Evans [46] caution against the use of universal cutoffs for Bayes factors, unless these are well calibrated. However, since we are using a model selection prior, we feel that the use of heuristic cutoffs recommended by Jarosz and Wiley [45] is acceptable for the purposes of this study.

In this paper, we report K compared to the null model (intercept only). Therefore, if Model 1 has $K_{1}=2.2\times 10^{55}$ and Model 2 has reported $K_{2}=1.1\times 10^{54}$ then both are overwhelmingly better than the null model. Furthermore, there is strong evidence in support of Model 1 compared to Model 2; ratio $K_{1}/K=20$.

For the fiducial models, there is no need to select a prior because the fiducial distribution is determined solely by the data and the model equation. Hannig et al. [36] show that the density of the generalized fiducial distribution is $r_{y}\;\left(\theta \right)=\frac{f\left(y|\theta \right)\;J\left(y,\theta \right)}{\int f\left(y|\theta \prime \right)\;J\left(y,\theta \prime \right)\;d\theta \prime },$ where f(x|θ) is the data likelihood and $J\left(y,\theta \right)=D\left(\nabla_{\theta }A\left(U,\theta \right){|}_{U=A^{-1}\left(y,\theta \right)}\right)$ with $\nabla_{\theta }$ being a gradient and $D\left(M\right)=\sqrt{det\left(M\prime M\right)}$ for any matrix *M*. Notice that this is similar to Bayesian posterior formula with the role of a prior π(θ) replaced by the Jacobian J(y,θ).

In order to compute the fiducial distribution, we need to select the data generating model equation. In this paper we use the data generating model equation

$$Y=A\left(U,\theta \right)=X\beta +{\left(\sigma_{a}^{2}\;S_{a}+\sigma_{e}^{2}\;I_{n}\right)}^{\frac{1}{2}}\;U,$$

where *Y* are the observed values of dependent variables ordered in a vector, the parameters $\theta \;=(\beta ,\sigma_{a}^{2},\sigma_{e}^{2}$) are the fixed effect coefficients, the variance of the random intercept and the variance of the residuals, respectively, *X* is the design matrix comprised of the predictors treated as fixed effects, *S_a_* is a n×n matrix with (*i*,*j*) entry equal to 1 if the observations *i* and *j* are on the same subject and 0 otherwise, *I_n_* is the *n* × *n* identity matrix, and *U* is a vector of independent standard Gaussian random variables. For this model, the Jacobian matrix simplifies to a matrix obtained by concatenation of columns

$$J\left(y,\theta \right)=\left[X,\;S_{a}{\left(\sigma_{a}^{2}\;S_{a}+\sigma_{e}^{2}\;I_{n}\right)}^{-1}\left(y-X\beta \right),{\left(\sigma_{a}^{2}\;S_{a}+\sigma_{e}^{2}\;I_{n}\right)}^{-1}\left(y-X\beta \right)\right].$$

The fiducial distribution is used to define confidence intervals for all parameters. As in the frequentist paradigm, we conclude a null effect for a parameter if the corresponding interval contains 0 and no values far away from 0, as in an equivalence test [20]. The advantage of generalized fiducial inference is that the confidence intervals are easily available for all parameters [21], while obtaining confidence intervals for the variance of the random parameters under the frequentist paradigm is difficult.

## 4. Results

Descriptive statistics for all study variables are displayed in Table 1. Analyses across the three statistical paradigms were conducted in R version 4.0.2. The frequentist results were produced using the package lmerTest version 3.1–2 [47] fitted using the default restricted maximum likelihood. The Bayesian analysis utilized the package BayesFactor 0.9.12–4.2 [48] using the default priors. The Fiducial analysis was done using a MCMC sampler RStan 2.21.2 [49]. The R code used to generate all results is included in the Supplementary Materials. The various models considered all follow the structure described in Section 3.2 differing only by which covariates are included. The lists of the included covariates for the various models are in Table 2.

An unconditional model was conducted to partition the variance in SBP-R within- and between-persons. Results showed that 51% (σ^2^ = 131.22, *SE* = 8.62) of the variance is within-person and 49% (τ_00_ = 125.97, *SE* = 35.79) of the variance is between-person, indicating sufficient variability at each level for subsequent analyses.

### 4.1. Frequentist Results

Results from each of the six models corresponding to each of the research questions are presented in Table 2.

**Figure 1:**
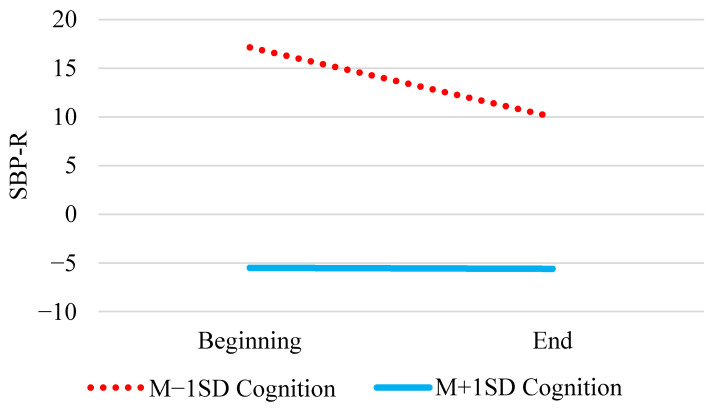
Cognition Differences in Changes in SBP-R.

Across all models, there was a significant and positive main effect of baseline SBP. In Model 1, there was a significant average decrease in SBP-R over time (γ_10_). People with higher cognition (γ_02_) as well as those with more physical health challenges (γ_03_) had lower SBP-R. Although changes in SBP-R did not differ by physical health challenges (γ_12_), changes in SBP-R did depend on cognition (γ_11_) (see Figure 1). SBP-R was stable for those with higher cognition (*b* = −0.11, *SE* = 0.14, *p* = 0.413), but SBP-R significantly decreased for those with lower cognition (*b* = −0.75, *SE* = 0.34, *p* = 0.027). There was a significant average decrease in SBP-R over time (γ_10_) in Model 2, as in Model 1. People with higher cognition (γ_02_) as well as those with more mental health challenges (γ_03_) had lower SBP-R. As in Model 1, we also found evidence of a Cognition × Tertile interaction (γ_11_) in Model 2 (Figure 1 also applies here). There was no evidence of mental health challenge differences in changes in SBP-R (γ_12_).

**Figure 2:**
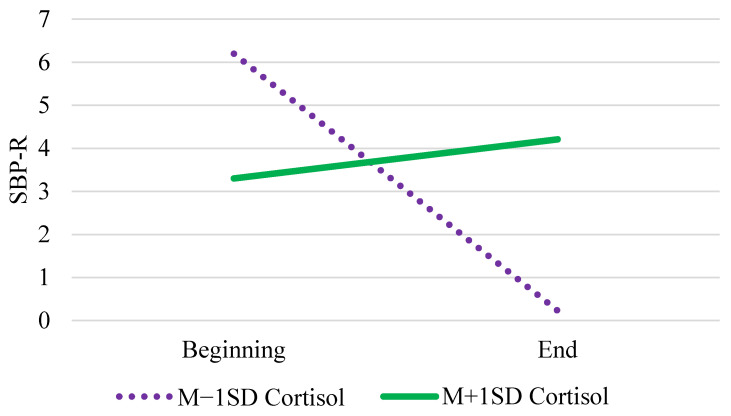
Cortisol Differences in Changes in SBP-R.

In Model 3, those with higher cognition (γ_02_) had lower SBP-R, and those differences predicted changes in SBP-R (γ_11_), as in the previous models. We found evidence of cortisol differences in SBP-R changes (γ_12_, see Figure 2), such that those with low cortisol experienced a significant decrease in SBP-R (*b* = −0.54, *SE* = 0.17, *p* = 0.001), whereas those with high cortisol did not experience significant changes (*b* = 0.14, *SE* = 0.08, *p* = 0.081).

**Figure 3:**
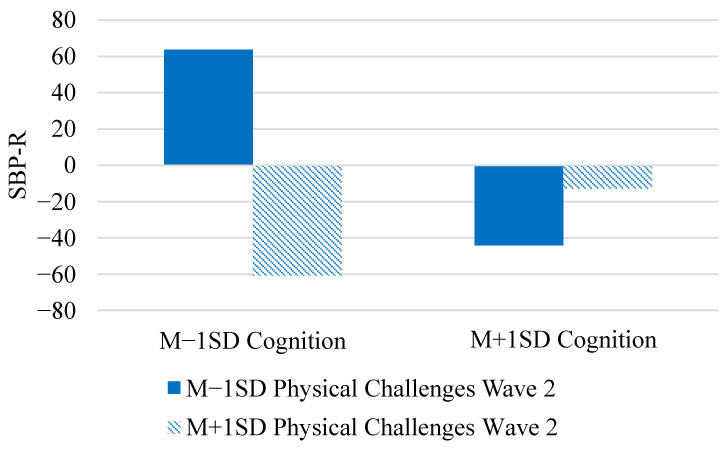
Changes in Physical Health Challenges Modifying the Relationship between Cognition and SBP-R.

Questions regarding changes in physical and mental health challenges and stress indicators were addressed in Models 4–6 (Table 2). There was a significant decrease in SBP-R (γ_10_) and those with higher cognition had lower SBP-R (γ_02_). Although cognition (γ_11_) and physical health challenges (γ_12_) did not predict changes in SBP-R, changes in physical health challenges from Wave 1 to Wave 2 did modify the association between cognition and SBP-R (γ_05_, see Figure 3). Changes in physical health challenges were associated with a bigger difference in SBP-R for those who were lower in cognition compared to those higher in cognition. Increases in physical health challenges from Wave 1 to Wave 2 for those low in cognition corresponded with the lowest SBP-R.

**Figure 4:**
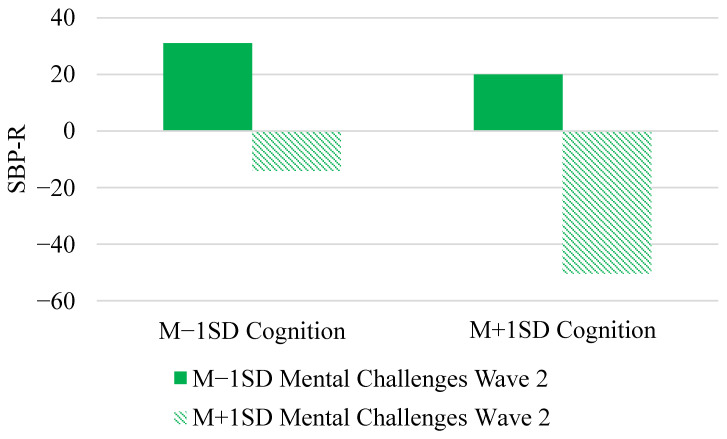
Changes in Mental Health Challenges Modifying the Relationship between Cognition and SBP-R.

Model 5 shows that those with high cognition (γ_02_), low mental health challenges at Wave 1 (γ_03_), and high mental health challenges at Wave 2 (γ_04_) had lower SBP-R. We also found evidence of an interaction where changes in mental health challenges from Wave 1 to Wave 2 did modify the association between cognition and SBP-R (γ_05_, see Figure 4). Changes in mental health challenges were associated with a bigger difference in SBP-R for those who were higher in cognition compared to those lower in cognition. Increases in mental health challenges from Wave 1 to Wave 2 for those higher in cognition corresponded with the lowest SBP-R.

**Figure 5:**
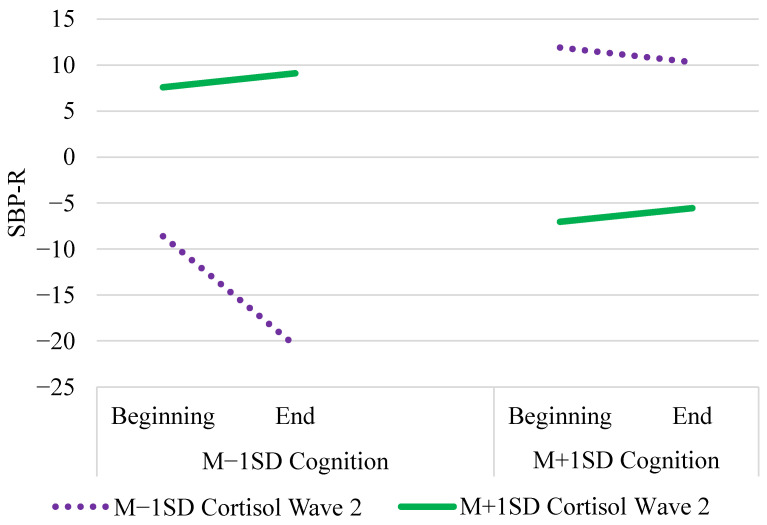
Changes in Cortisol Modifying Cognition Differences in Changes in SBP-R (Cortisol × Cognition × Tertile).

Model 6 shows that those with higher cognition reported lower SBP-R (γ_02_). We also found evidence of two 2-way interactions (Tertile × Cortisol, γ_12_; Cognition × Cortisol, γ_05_) which were qualified by a significant 3-way interaction (Tertile × Cortisol × Cognition, γ_13_; see Figure 5). The steepest decrease in SBP-R was for those with low cognition who experienced a decrease in cortisol from Wave 1 to Wave 2.

### 4.2. Bayesian Results

We conducted six analyses to address the six research questions, with the same fixed and random effects shown in each of the models in Table 2. As noted above, a model with only the intercept included was compared to all possible subsets (26 for each of the first three models corresponding with Models 1–3, 76 for each of the last three models corresponding with Models 4–6) of the relevant fixed effects. For the first question regarding cognition and physical health challenges predicting changes in SBP-R over time, the best fitting model included SBP at baseline and main effects of tertile, cognition, and physical health challenges (K = 8.73 × 10^53^). However, the model that included the above effects plus the addition of the cross-level interaction between cognition and tertile (K = 7.75 × 10^53^ see Figure 1) was only slightly less likely than the best fitting model (the ratio of the Bayes factor comparing the best fitting model with the model that includes the interaction is 0.89). Similar results were found for the models regarding cognition and mental health challenges. The best fitting model included SBP at baseline and main effects of cognition and mental health challenges (K = 1.64 × 10^62^), but the model that included the cross-level interaction between cognition and tertile (K = 5.53 × 10^61^) was ⅓ as likely as the best fitting model. With respect to the question addressing cortisol differences in changes in SBP-R, the best fitting model only included SBP at baseline and a main effect of cognition (K = 3.97 × 10^55^). The model that included the best fitting effects plus tertile, cortisol, Cognition × Tertile, and Cortisol × Tertile (K = 8.86 × 10^54^), was somewhat unlikely (0.22), indicating that the evidence is not strong enough to claim a null effect, but that the Cortisol × Tertile interaction (Figure 2) should be interpreted with some caution.

The models examining the effects of changes in physical health challenges, mental health challenges, and cortisol from Wave 1 to Wave 2 each included 76 possible subsets compared to the intercept only model. For the model examining physical health challenges, the best fitting model contained main effects of SBP at baseline, cognition, physical health challenges at Wave 2, and the interaction between cognition and physical health challenges at Wave 2 (K = 2.37 × 10^49^, see Figure 3). The next best fitting model included the terms above as well as physical health challenges at Wave 1 (K = 1.33 × 10^49^) and was 0.56 as likely as the best fitting model. With respect to mental health challenges, the best fitting model contained SBP at baseline, cognition, mental health challenges at Wave 1, mental health challenges at Wave 2, and the interaction between cognition and mental health challenges at Wave 2 (K = 1.32 × 10^27^, see Figure 4). The final model examined cognition and changes in cortisol differences in SBP-R over time. The best fitting model included SBP at baseline, cognition, cortisol at Wave 1, cortisol at Wave 2, and the interaction between cognition and cortisol at Wave 2 (K = 1.80 × 10^34^). The model that contained the 3-way interaction (Figure 5) was 0.01 as likely as the best fitting model, so we interpret this interaction with some caution.

### 4.3. Fiducial Results

Results for the six multilevel fiducial models are presented in Table 2. With one minor exception of the main effect of tertile (γ_10_) in Model 4, the pattern of results for the fixed effects replicated all of the results from the frequentist models. However, the fiducial models provide more information (i.e., confidence intervals for all parameters, including random effects) and have the benefit of the fiducial distribution which does not rely on a prior (as in Bayesian models) but instead derives the information from the data.

## 5. Discussion

Our primary goal was to demonstrate the utility of frequentist, Bayesian, and fiducial methods for evaluating the robustness of factors that may contribute to cognitive engagement for older adults experiencing cognitive decline. In doing so, we developed fiducial methods, implemented them in R and STAN, and made the code available as a Supplementary document.

### 5.1. Findings That Converge across the Paradigms

All three statistical paradigms suggested evidence for the presence of cognition differences in changes in SBP-R. With normal aging, we would expect increases in difficulty to correspond to increases in SBP-R, reflecting increased engagement [1], a pattern which was observed in a study using a slightly longer version of the task in the present study [3]. In our cognitively impaired sample, we found that those who were less cognitively impaired (i.e., *M* + 1*SD* cognition in Figure 1) did not experience an increase or decrease in SBP-R. In line with selective engagement theory [1], relative maintenance of SBP-R levels among the low-impaired individuals may reflect a lack of motivation, as the benefits of engaging in laboratory tasks may not outweigh the required cognitive costs, which are higher than those experienced by cognitively healthy individuals. Highly impaired people (i.e., *M* − 1*SD* cognition) experienced significant decreases in SBP-R, reflecting disengagement with the task.

We also examined SBP-R throughout the task among those with low versus high cortisol levels (Figure 2), finding a pattern of decreasing SBP-R for those with low HCC levels, but maintained SBP-R with a slight increase throughout the task, although not statistically significant, for those with high HCC levels. These findings should be interpreted with some caution, as the Bayesian analysis did not strongly support this model. Decreasing SBP-R among those with low cortisol may indicate disengagement from the task, whereas the slight nonsignificant increase in SBP-R among those with high cortisol may reflect attempts to increase effort as task difficulty increased, the latter being a pattern expected in healthy aging (e.g., [3]). Cortisol may serve as a protective factor towards the end of life [50], helping to mobilize limited resources. These protective effects may extend to cognitive engagement as well. 

We found that changes in physical health from Wave 1 to Wave 2 were more consequential for those with high levels of cognitive impairment than for those with more mild impairments (Figure 3). Highly impaired individuals who experienced decreases in their physical health challenges (i.e., became healthier) exhibited relatively high levels of SBP-R, whereas highly impaired individuals who experienced increases in their physical health challenges (i.e., became less healthy) exhibited relatively low levels of SBP-R. We view the especially low levels of SBP-R among those experiencing health decline in the context of high cognitive impairment as representative of compounding vulnerabilities [10]. Additionally, the finding that physical health improvements corresponded with overall higher SBP-R among highly impaired individuals may support the contention that hypertension among the oldest–old has benefits for cognition and health [7]. We view this effect as representative of reactivity to the cognitive stressor, rather than the ability to remain (or increase) engagement over time within the task because we do not find evidence of an interaction with tertile.

Whereas the previous model suggested that physical health changes were most influential for those who are highly impaired, Figure 4 shows that mental health changes had the strongest effects for mildly impaired individuals. Among those with lower levels of cognitive impairment, increases in mental health challenges predicted higher SBP-R, whereas decreases in mental health challenges predicted lower SBP-R. Mental health challenges have been associated with low motivation for cognitive engagement in healthy older adults [3]. One interpretation is that the effects of mental health on motivation are similar among those with mild impairment, whereas highly impaired individuals may not respond to changes in mental health in a similar way. Early in the dementia process, the emotional effects of cognitive decline are powerful as individuals become aware of their decline and experience related anxiety and fear [51]. The beginning stages of cognitive decline may represent a sensitive period of development, during which emotional challenges are particularly consequential for motivation to engage in demanding activities which may be beneficial and necessary for determining the rate of future decline. Our findings suggest that declining mental health may relate to motivation for cognitive engagement only when the cognitive resources are available to assess aspects of the situation.

However, it is important to note that we found strong evidence of a null effect of mental health challenges predicting changes in SBP-R across all three statistical paradigms. *Changes* in mental health may be associated with motivation (as we note above) more so than the *level* of mental health challenges. Thus, we suggest that within-person processes with respect to mental health changes may be more important than between-person differences.

### 5.2. Findings that Diverge across the Paradigms

We found somewhat mixed evidence for the simultaneous effects of cognitive ability, long-term changes in cortisol, and time throughout the task as predictors of SBP-R. Both the frequentist and fiducial analyses found a three-way interaction in which individuals high in cognitive impairment who experienced long-term increases in cortisol showed decreases in SBP-R throughout the task (Figure 5). Although the Bayesian analysis provided minimal support for this finding, these results are heavily influenced by the prior, which is potentially why the results diverge from the frequentist and fiducial results. Therefore, we interpret the three-way interaction with caution, but suggest that this pattern may represent additional evidence for the protective effects of cortisol among those high in cognitive impairment [52]. Individuals high in cognitive impairment appear to have patterns of SBP-R similar to cognitively healthy older adults (i.e., slight increases throughout the task which may be interpreted as appropriate increases in effortful engagement). Thus, increases in cortisol among highly impaired older adults may serve a compensatory role.

There was also a point of disagreement between the frequentist and fiducial paradigms with respect to the main effect of tertile (Model 4). Whereas the *p* value (.0475) in the frequentist paradigm would lead us to conclude that there was a significant decrease in SBP-R over time, the fiducial confidence interval contained 0. In this instance, the fiducial paradigm provides a more conservative estimate of the effect, perhaps highlighting the somewhat arbitrary nature of *p* value cut-offs [22].

Given the observed areas of convergence and divergence, we suggest some preliminary steps for researchers when selecting paradigms. For hypotheses with very strong priors that researchers are confident are correct, the Bayesian approach is a viable option [21]. However, if priors are difficult to ascertain, as we believe they are within repeated-measures data from cognitively impaired samples, we assert that the fiducial paradigm would be a good compromise between Bayesian and frequentist. When investigating developmental phenomena without clear expectations based on previous work or theory, there can be tremendous value in applying all three statistical paradigms. If there is a clear disagreement among the three methods, we would caution against putting too much trust in any one of them. Clear disagreement could suggest that the underlying different assumptions made by the various approaches have a strong effect on the result. It may also be useful to consider sample size when selecting a paradigm. Because Bayesian and fiducial inference are not based on asymptotic distributions as most tests in multilevel models are, they are especially useful for studies with relatively small sample sizes. If an appropriate prior is available within the Bayesian paradigm, some uncertainty can be reduced by incorporating prior information, thus maximizing the value of the small sample. With respect to fiducial inference, where a prior is not necessary, bias is not introduced by incorporating inappropriate or inaccurate information which could unduly influence the results of a small sample.

### 5.3. Limitations and Future Directions

The findings of the current study should be considered alongside some limitations. We note that our multilevel models are more complex than currently available code to determine power [53], which is based on the frequentist paradigm only, so we were unable to obtain reliable estimates of power for our models. Our rigorous testing of each model across three paradigms along with up to 24 observations per person helps to address this issue, but we acknowledge that a larger person level sample size would be ideal. In addition, selecting the prior for the Bayesian models is a complex decision tree [21] and it is possible that applying different decision rules would result in a different prior, which may alter the Bayesian results. Whereas a clear strength of the fiducial paradigm is the existence of confidence intervals for random effects, priors for multilevel random effects in the Bayesian paradigm are still being developed so a careful examination of appropriate priors is an important future direction.

## 6. Conclusions

By computing frequentist, Bayesian, and fiducial multilevel models, we detected areas of convergence as well as divergence with respect to predictors of cognitive engagement in cognitively impaired older adults. Our findings extend the principles of selective engagement theory [1] to cognitive impairment, as limited cognitive resources may result in reliance on other types of resources. Our results highlight the importance of health challenges and stress as moderators. Long-term changes in mental health may be particularly important at the beginning of cognitive decline, and increases in physical health challenges over time were associated with the lowest levels of engagement in those who were highly impaired. In addition, high levels of cortisol and/or long-term increases in cortisol may serve a compensatory role for those with high levels of cognitive impairment. These findings were mostly consistent across the three statistical paradigms, providing us additional confidence in determining effects. Further, the Bayesian and fiducial paradigms can be especially useful for relatively small sample sizes because they are not based on asymptotic distributions. In particular, the fiducial paradigm is a useful tool because it provides more information than *p* values without the need to specify prior distributions, which may unduly influence the results based on a small sample.

## Figures and Tables

**Table 1:** Descriptive Statistics (Between-Person) of Study Variables.

Variables	*M*	*SD*
SBP-R	4.41	11.56
SBP at baseline	130.92	21.31
Cognition Factor	−0.03	0.90
Recall (HCAP)	3.90	1.88
Serial Subtraction (HCAP)	3.56	1.80
CSI-D	7.80	1.38
Letter-Number Sequencing	8.42	3.04
Digit-Symbol Substitution	46.30	15.71
Plus-Minus Task	69.64	74.22
Stroop Task	36.30	24.01
Short Blessed	8.58	4.45
Mental Health Challenges	0.01	0.68
Geriatric Depression Scale	0.89	1.44
SF-36 Mental	48.36	11.54
Physical Health Challenges	−0.04	0.67
Number of chronic conditions	3.58	2.18
SF-36 Physical	46.51	6.47
Cortisol (log)	2.35	1.47

Note. Cognition Factor, Mental Health Challenges, and Physical Health Challenges are factor scores of the variables indented under each.

**Table 2:** Unstandardized Estimates, Standard Errors, and 95% Confidence Intervals Predicting Systolic blood pressure responsivity (SBP-R) using Frequentist and Generalized Fiducial Inference (GFI) Multilevel Models.

Variable	Parameter	Frequentist Estimates	GFI Estimates	95% GFI CI
Model 1				
Intercept	γ_00_	5.82 (3.65)	5.83 (3.87)	(−1.92, 13.30)
Tertile	γ_10_	−0.33 * (0.15)	−0.33 (0.15)	(−0.64, −0.03)
SBP at baseline	γ_01_	0.22 ***(0.06)	0.22 (0.07)	(0.09, 0.35)
Cognition	γ_02_	−11.82 (2.57)	−11.98 (3.16)	(−18.28, −6.11)
Physical health challenges	γ_03_	−6.59 * (2.57)	−6.53 (2.60)	(−11.65, −1.48)
Tertile × Cognition	γ_11_	0.32 * (0.16)	0.32 (0.16)	(0.01, 0.64)
Tertile × Physical Health	γ_12_	0.13 (0.22)	0.13 (0.21)	(−0.29, 0.56)
Between-person variance	τ_00_	318.9 (17.86)	356.37	(160.57, 720.52)
Within-person variance	σ^2^	121.5 (11.02)	122.02	(105.97, 140.45)
Model 2				
Intercept	γ_00_	5.34 (5.42)	5.31 (5.71)	(−5.89, 16.70)
Tertile	γ_10_	−0.31 * (0.14)	−0.31 (0.14)	(−0.58, −0.04)
SBP at baseline	γ_01_	0.22 *** (0.06)	0.22 (0.06)	(0.10, 0.33)
Cognition	γ_02_	−23.33 *** (2.68)	−23.27 (3.17)	(−29.33, −16.99)
Mental health challenges	γ_03_	−23.57 *** (3.13)	−23.58 (3.35)	(−30.08, −16.96)
Tertile × Cognition	γ_11_	0.36 * (0.15)	0.36 (0.15)	(0.07, 0.66)
Tertile × Mental Health	γ_12_	0.15 (0.20)	0.15 (0.20)	(−0.25, 0.53)
Between-person variance	τ_00_	742.2 (27.24)	808.12	(384.35, 1542.38)
Within-person variance	σ^2^	103.1 (10.15)	103.76	(90.12, 119.77)
Model 3				
Intercept	γ_00_	4.74 (3.38)	4.76 (3.54)	(−2.19, 11.89)
Tertile	γ_10_	−0.23 (0.15)	−0.23 (0.15)	(−0.53, 0.06)
SBP at baseline	γ_01_	0.21 *** (0.05)	0.22 (0.06)	(0.11, 0.33)
Cognition	γ_02_	−10.12 ***(2.32)	−10.20 (3.16)	(−15.62, −4.99)
Cortisol	γ_03_	−1.08 (1.16)	−1.07 (1.20)	(−3.40, 1.25)
Tertile × Cognition	γ_11_	0.38 * (0.16)	0.38 (0.16)	(0.06, 0.69)
Tertile × Cortisol	γ_12_	0.22 * (0.10)	0.22 (0.10)	(0.01, 0.42)
Between-person variance	τ_00_	260.2 (16.13)	289.03	(135.09, 576.38)
Within-person variance	σ^2^	121.5 (11.02)	122.02	(106.42, 139.90)
Model 4				
Intercept	γ_00_	−11.56 (17.21)	−12.14 (20.26)	(−52.43, 27.09)
Tertile	γ_10_	−0.37 * (0.19)	−0.37 (0.19)	(−0.75, 0.004)
SBP at baseline	γ_01_	0.74 *** (0.06)	0.75 (0.06)	(0.62, 0.87)
Cognition	γ_02_	−15.99 *** (3.01)	−16.02 (3.04)	(−22.02, −0.10)
Physical challenges Wave 1	γ_03_	13.00 (35.49)	13.39 (42.37)	(−67.28, 96.14)
Physical challenges Wave 2	γ_04_	−28.45 (29.14)	−29.09 (34.88)	(−96.27, 36.09)
Tertile × Cognition	γ_11_	0.04 (0.24)	0.04 (0.24)	(−0.43, 0.51)
Tertile × Physical Wave 2	γ_12_	0.02 (0.24)	0.02 (0.24)	(−0.44, 0.49)
Cognition × Phys. Wave 2	γ_05_	51.37 *** (4.38)	51.39 (4.46)	(42.60, 60.15)
Tertile × Cog. × Phys. w2	γ_13_	0.40 (0.30)	0.40 (0.30)	(−0.19, 0.99)
Between-person variance	τ_00_	2603.9 (51.03)	3482.92	(1127.96, 9974.09)
Within-person variance	σ^2^	100.3 (10.02)	101.26	(84.28, 121.27)
Model 5				
Intercept	γ_00_	−1.68 (6.14)	−1.74 (7.13)	(−16.20, 12.70)
Tertile	γ_10_	−0.31 (0.25)	−0.31 (0.25)	(−0.81, 0.50)
SBP at baseline	γ_01_	0.35 *** (0.07)	0.35 (0.08)	(0.20, 0.87)
Cognition	γ_02_	−14.02 ** (4.26)	−14.08 (4.53)	(−23.19, −5.21)
Mental challenges Wave 1	γ_03_	48.08 * (19.32)	47.43 (22.18)	(4.2887, 91.98)
Mental challenges Wave 2	γ_04_	−35.44 * (14.73)	−34.94 (16.78)	(−69.057, −2.80)
Tertile × Cognition	γ_11_	0.14 (0.31)	0.14 (0.31)	(−0.46, 0.74)
Tertile × Mental Wave 2	γ_12_	−0.04 (0.30)	−0.05 (0.30)	(−0.64, 0.55)
Cognition × Ment.Wave 2	γ_05_	−7.55 * (3.65)	−7.55 (3.70)	(−14.63, −0.22)
Tertile × Cog. × Ment. w2	γ_13_	0.21 (0.34)	0.21 (0.34)	(−0.47, 0.89)
Between-person variance	τ_00_	371.2 (19.27)	501.73	(146.46, 1464.99)
Within-person variance	σ^2^	176.5 (13.28)	178.25	(148.90, 212.98)
Model 6				
Intercept	γ_00_	−5.79 (9.29)	−5.86 (10.35)	(−26.25, 14.53)
Tertile	γ_10_	0.22 (0.25)	0.23 (0.25)	(−0.26, 0.72)
SBP at baseline	γ_01_	0.43 *** (0.06)	0.44 (0.07)	(0.31, 0.57)
Cognition	γ_02_	−11.31 *** (3.10)	−11.29 (3.18)	(−17.66, −5.11)
Cortisol Wave 1	γ_03_	−8.59 (8.64)	−8.43 (9.62)	(−28.48, 10.68)
Coritsol Wave 2	γ_04_	−6.09 (10.87)	−6.34 (12.07)	(−30.29, 17.62)
Tertile × Cognition	γ_11_	−0.02 (0.25)	−0.02 (0.25)	(−0.51, 0.48)
Tertile × Cortisol Wave 2	γ_12_	0.79 * (0.34)	0.80 (0.34)	(0.13, 1.46)
Cognition × Cort. Wave 2	γ_05_	24.10 *** (3.95)	24.32 (4.10)	(16.33, 32.35)
Tertile × Cog. × Cort. w2	γ_13_	−0.83 * (0.38)	−0.84 (0.38)	(−1.58, −0.09)
Between-person variance	τ_00_	928.7 (30.47)	1146.28	(429.03, 2817.93)
Within-person variance	σ^2^	132.5 (11.51)	133.46	(113.88, 156.87)

Table Note. * *p* < 0.05, ** *p* < 0.01, *** *p* < 0.001 for frequentist estimates. SBP = systolic blood pressure, Phys. = Physical health challenges, Ment. = Mental health challenges, Cort. = Cortisol, w2 = Wave 2.

## Data Availability

The data presented in this study are available on request from the corresponding author.

## Supplementary Materials

The following are available online at https://www.mdpi.com/article/10.3390/e23040428/s1, The R code used to generate all results is included in the supplemental materials.

## Appendix A

### Appendix A.1. Additional Participant Information

Individuals were recruited through local newspaper advertisements and senior citizen community organizations in the Raleigh, North Carolina area, and were excluded if they did not have a relevant diagnosis, if they did not fail the cognitive screener, or if they or a family member reported them being unable to sit still for an extended period of time. At Wave 1, the average age of the 28 participants was 72.14 (*SD* = 6.20), 16 were women, 23 self-identified as White, 3 self-identified as Black or African American and 1 self-identified as Asian. Twenty earned a college education or higher, 9 reported having a diagnosis of dementia or cognitive impairment, and 7 had a family member or caregiver to serve as a proxy with them during the study session. Of the 28 participants, 14 participated in the Wave 2 study approximately 6 months later before in-person testing was halted due to the COVID-19 pandemic. Complex data collection procedures with intensive physiological assessments typically rely on relatively small sample sizes (e.g., [54]), and here we focus on the large number of within-person assessments. Participants received $50 for compensation at each of the two waves of the study.

### Appendix A.2. Additional Measures Information

#### Appendix A.2.1. Physical Health Challenges

A chronic condition checklist similar to that used in the Midlife in the United States study (http://midus.wisc.edu, accessed on 5 October 2018) was used to assess number of illness or health problems participants were currently dealing with, and the SF-36 Physical Component Summary score [55] was used to assess subjective physical health. These items were combined to create a factor score representing physical health challenges, with higher values indicating worse physical health.

#### Appendix A.2.2. Mental Health Challenges

The Geriatric Depression Scale [56] was used to assess number of depressive symptoms and the SF-36 Mental Component Summary score [55] was used to assess mental health. A factor score representing mental health challenges was created from these items, with higher values indicating worse mental health.

#### Appendix A.2.3. Cortisol

To assess cumulative cortisol levels over the three months preceding lab visits, we collected hair samples following procedures outlined by Stalder and Kirschbaum [20] and detailed below in the procedure section. Samples were sent to Clemens Kirschbaum’s lab in Dresden, Germany, for immunoassay of cortisol. Results were received in an Excel spreadsheet with HCC values reported in pg/mg. Consistent with previous work [20], we conducted a log transformation of the HCC values for analysis. As described in a meta-analysis [57], HCC is a valid measure across studies, with consistently higher average levels in groups with chronic stress.

#### Appendix A.2.4. Cognitive Ability Assessment

Participants completed a wide range of cognitive assessments. The Short Blessed [39] was administered over the telephone and used to assess potential cognitive impairment. General cognitive impairment was also assessed in the lab using the Community Screening Instrument for Dementia [58]. The remaining assessments measured specific areas of cognition. Memory was assessed using a modified version of the immediate and delayed word recall tests from the Health and Retirement Study 2016 Harmonized Cognitive Assessment Protocol [59]. Working memory was assessed using serial subtractions from the Harmonized Cognitive Assessment Protocol [59] and the letter-number sequencing test from the Wechsler Adult Intelligence Scale, Third Edition (WAIS-III) [60]. Processing speed was measured using the digit-symbol substitution test (WAIS-III) [60]. Task-switching costs were assessed using the plus-minus task [61,62], which consists of three parts: addition problems, subtraction problems, and alternating between the two types of arithmetic problems. Inhibitory control was assessed using a Stroop test, involving three parts: naming the colors of a series of blocks, reading a list of colors printed in black ink, and naming the colors of ink used to print a list of colors incongruent with the ink color. For all tasks, participants were reminded of the instructions as necessary. Scores on these tests were combined to create a factor score with higher values indicating higher cognitive ability, or less cognitive impairment.

### Appendix A.3. Additional Procedure Information

The procedure is a modified version of a similar study of engagement in a cognitively normal sample of older adults e.g., [3]. All participants gave their informed consent for inclusion before they participated in the study. The study was conducted in accordance with the Declaration of Helsinki, and the protocol was approved by the North Carolina State University IRB (protocol #5545).

The length of 3 cm of hair for the cortisol assessments was chosen because it captures a 3-month span (1 cm/month). Hair samples were collected as close to the scalp as possible in the posterior vertex region of the head. It is essential to cut close to the scalp for the most “up-to-date” cortisol levels [63]. For long hair samples, the sample was sectioned carefully from the rest of the hair with embroidery floss. If the participant had hair too short to cut a 3cm sample, we cut along a clear line in the same scalp region and collected the hair in a pouched envelope. Participants were asked questions regarding their hair care maintenance including how many times a week they washed their hair, if their hair had been recently bleached, if they use conditioner, or if they had recently had a perm. Answers to these questions were documented but none were exclusionary. Once the hair samples were collected, they were sealed in foil for preservation.
